# Parents' attitudes towards topical fluoride and vaccines for children: Are these distinct or overlapping phenomena?

**DOI:** 10.1016/j.pmedr.2018.02.014

**Published:** 2018-03-06

**Authors:** Richard M. Carpiano, Donald L. Chi

**Affiliations:** a School of Public Policy, University of California, Riverside, Riverside, CA, USA; b Department of Sociology, University of California, Riverside, USA; c Center for Healthy Communities, University of California, Riverside, USA; d Department of Sociology, University of British Columbia, Vancouver, BC, Canada; e Department of Oral Health Sciences, School of Dentistry, University of Washington, Seattle, WA, USA

**Keywords:** Vaccinations, Topical fluoride, Vaccination refusal, Fluoride refusal, Vaccine hesitancy, Fluoride hesitancy, Children, Adolescents, Preventive treatment, United States

## Abstract

Despite attention paid to parental refusal of child vaccines, the phenomenon of topical fluoride refusal is poorly understood. We examine the extent to which parent attitudes and Internet use regarding topical fluoride treatment and vaccines may overlap and, in turn, uniquely or distinctly correlate with fluoride and vaccine refusal for the child. In 2017, we analyzed data collected from 2011 to 12 for 361 children from three Washington state dental clinics. The instrument included analogous measures of topical fluoride and vaccine safety concerns, perceived severity of preventable cavities/disease, and Internet use for fluoride/vaccine information; and measures of non-fluoridated toothpaste use, attitudes towards dental x-rays and amalgam and composite fillings. We assessed dental chart-based topical fluoride refusal occurring in 2009 or 2010 and parent-reported vaccine refusal. All analogous fluoride and vaccine items were substantively correlated. However, in a series of adjusted models, none of these items were significantly associated with fluoride refusal. Multiple fluoride and vaccine items were associated with vaccine refusal in unadjusted models; but only vaccine safety concerns, perceived severity of a preventable cavity, and Internet use for vaccine information remained significant in adjusted models. Although there is concordance between the two refusal behaviors as well as analogous attitudes and Internet use, these findings challenge the idea that fluoride refusal should be addressed with interventions focusing on vaccine refusal. Further research is required on the factors underlying refusal of preventive dental care.

## Highlights

• Studies find that parental refusal of child topical fluoride and vaccines correlate.
• We tested overlap of factors possibly underlying refusal of both treatments.
• Analogous fluoride and vaccine attitudes and Internet use items correlated.
• None of these items were significantly associated with fluoride refusal.
• Few vaccine- and fluoride-related items were associated with vaccine refusal.

## 1 Introduction

Vaccine-preventable disease outbreaks in the US and internationally have led to increased attention towards understanding and addressing vaccine hesitancy among parents. Parental determinants of vaccine hesitancy for their children include concerns about vaccine safety (including fear for adverse events), anticipated feelings of regret or guilt if the child contracts a vaccine-preventable disease or suffers from an adverse event, and using the Internet to search for information (Dubé et al., 2013; Salmon et al., 2015).

Do parents' vaccine attitudes indicate similar attitudes and refusal of other types of preventive care, including preventive dental care? Numerous studies have identified how health attitudes, norms, and behaviors cluster and constitute individualistic and collective health lifestyles (Abel, 1991; Cockerham, 2005; Slater and Flora, 1991), yet little attention has been paid to how vaccination attitudes and refusal potentially cluster with other health domains. Such clustering may reflect more latent orientations towards treatments viewed as more “natural” and thus safer; or even broader dimensions of parenting, including “intensive parenting” practices that heavily emphasize managing a child's potential health and developmental risks (Reich, 2016).

Recent evidence indicates that vaccine refusal correlates with topical fluoride treatment, a type of preventive dental care regularly provided at a dental office and also offered at medical clinics (Chi, 2014). However, the extent to which refusal of these two types of preventive care reflect common attitudes and behaviors is unclear.

The present study contributes to this knowledge gap via a two-step analysis of topical fluoride and vaccination attitudes, behaviors, and refusal. First, we examine the extent of convergence between parent attitudes and behaviors regarding topical fluoride treatment and child vaccines. Specifically, we assess three parallel factors regarding vaccination and fluoride: (1) concern about safety and risks, (2) perceived severity if the child were to develop a disease or cavity that could have been respectively prevented by vaccination or fluoride—based on constructs of the Health Belief Model (Strecher and Rosenstock, 1997) and Extended Parallel Process Model (Askelson et al., 2015)—and (3) Internet use to obtain information about each preventive treatment (Seymour et al., 2015). To further elucidate whether vaccine and/or fluoride attitudes reflect more underlying beliefs about medical and dental treatments, we also examine how these three domains correlate with attitudes about three other dental procedures (x-rays, amalgam and composite fillings) and use of fluoride toothpaste.

Second, we consider to what extent these abovementioned fluoride- and vaccine-specific attitudes and behaviors correlate with both refusal behaviors. Empirically testing these parental attitudes' and behaviors' relative associations with topical fluoride and vaccine refusal for children allows us to evaluate the degree to which these two refusal behaviors reflect common or unique attitudes and behaviors. The answers to these questions will provide important insight on how to address refusal behaviors in clinical settings.

## 2 Methods

### 2.1 Study sample

Our data come from a case control study of parental refusal of topical fluoride for their children, conducted in 2011–12 among patients and their parents from three dental clinics in Washington state. Specific details regarding data collection and questionnaire have been previously reported (Chi, 2014), but briefly, those surveyed included parents whose child was seen for a dental checkup in one of the three study clinics in 2009 or 2010 (N = 1024). An English-language pre-tested survey was administered to parents who refused topical fluoride for their child (based on information from the child's dental records) and those who did not. Cases and controls were matched 1:1 on topical fluoride status, clinic, age, and gender. Surveys were mailed to parents with a $2 incentive included. Additional phone and repeat mailing attempts were made to collect data from non-responsive parents. The University of British Columbia and University of Washington research ethics boards reviewed and approved this study.

Of the 361 parents in the sample, 277 (76.7%) had complete (non-missing) information on all variables in this study. Item-specific missingness ranged from 0 to 7.4%. When such an overall level of missingness exists, complete case analysis is not recommended due to the potential for introducing bias and reducing statistical power (Schafer, 1999). Hence, we used the imputation by chained equations (ICE) method in Stata 13’s multiple imputation (MI) module (StataCorp, College Station, Texas) to estimate plausible values for all missing values. ICE entails specifying a series of multivariable equations to estimate multiple plausible values for each missing value (White et al., 2011). This procedure leads to the creation of a series of *m* datasets, each of which contains the actual values for all complete/non-missing observations and an imputed value for each missing observation. Based on recommendations in the MI literature, we computed *m* = 25 different datasets to ensure adequate variability in plausible values (White et al., 2011). Stata computes all analyses separately on each of these 25 datasets of n = 361 cases and then aggregates the results based on Rubin's method into one final set of estimates (Schafer, 1999). Our MI-based results revealed the same pattern of findings and substantive conclusions as complete case analysis.

### 2.2 Measures

*Topical fluoride and vaccine refusal* were both binary variables (coded 1 = refusal, 0 = accepted), respectively based on chart records and parental self-report regarding ever refusing to have their child immunized. In the survey, caregivers were asked whether they had ever refused topical fluoride for the children in their care at a dental care visit.

Perceived fluoride and vaccine side effects/safety were each based on the mean of two items. *Fluoride concern* items assessed how concerned the parent is that (1) her/his child might have a serious side effect from topical fluoride provided at the physician's or dentist's office and (2) the topical fluoride her/his child receives at the physician's or dentist's office might not be safe. *Vaccine concern* items analogously assessed how concerned the parental is that (1) her/his child might have a serious side effect from a shot and (2) childhood shots might not be safe. All four items were coded on a four-point scale from “not at all concerned” = 0 to “very concerned” = 3. The correlations for the fluoride-specific (r = 0.74) and vaccine-specific (r = 0.77) items respectively indicated a high degree of item consistency for each scale.

*Perceived severity of potential disease* consisted of two items asking the parent how bad it would be if the child got (1) a cavity preventable by fluoride and (2) one of the diseases that shots might have prevented. Both items were coded on a four-point scale from “horrible for my child” = 0 to “not that bad for my child” = 3.

*Internet use for fluoride/vaccine information* consisted of two binary measures (yes = 1; no = 0) respectively asking the responding parent if s/he uses the Internet to help decide whether her/his child gets topical fluoride and “shots.”

Disapproval of dental treatments consisted of four items: three asking parents how “OK” they are with dental x-rays, amalgam (“silver colored”) fillings, and composite (“tooth colored”) fillings for the child (each coded okay = 0; somewhat or not okay = 1), and fluoride toothpaste use by the child (coded as does use = 0 and does not use and [n = 17] “don't know” = 1).

Demographic covariates included parents' age, education, annual family income, and dental insurance status; child's sex, age (computed from date of birth and date survey was returned), race (white versus non-white), and Hispanic ethnicity; and clinic site.

### 2.3 Statistical analyses

Our analyses in 2017 proceeded in two steps. First, we computed bivariate correlations to determine the convergence between analogous fluoride- and vaccine-related variables for the abovementioned constructs. From a psychometric standpoint, this requires focusing on the magnitude of the correlations to determine substantive (versus statistical) significance. Furthermore, Stata user-created programs to compute correlations from MI data do not estimate *p*-values. Second, we examined the extent to which vaccine and fluoride-related and dental procedure variables were associated with refusal by estimating a series of Poisson regression models (with robust standard errors) that included demographic covariates, reporting prevalence ratios and 95% confidence intervals. These robust Poisson models produced results similar to those obtained using binary logistic regression models, but enabled reporting results as probabilities versus odds (Barros and Hirakata, 2003).

## 3 Results

Table 1 reports descriptive statistics for our study variables. Our sample was diverse with respect to demographic factors (e.g., socioeconomic status, parent and child age, race-ethnicity). Notably, 85.2% of parents reported having dental insurance.

**Table 1:** Imputed descriptive statistics of study variables (n = 361).^a^

	%	Empirical range
Parent and child sociodemographics		
Education		
≤ High school	23.7	0–1
Some college	32.5	0–1
4-Year college graduate or more	43.8	0–1
Income		
$20,000 or less	23.8	0–1
>$20,000 to 40,000	24.6	0–1
>$40,000 to 60,000	14.4	0–1
>$60,000	37.2	0–1
Parents' age (years)		
≤35	23.3	0–1
36–50	61.6	0–1
≥51	15.2	0–1
Child's sex is female	46.6	0–1
Child's age, mean	11.14 (0.26)	2.33–20.12
Child's race/ethnicity		
White (vs. non-white)	52.4	0–1
Hispanic ethnicity (vs. non-Hispanic)	14.6	0–1
Dental insurance	85.2	0–1
Clinic type		
University-based pediatric dentistry clinic	62.9	0–1
Community-based pediatric dentistry clinic	16.6	0–1
Private practice dentistry clinic	20.5	0–1

Refusal		
Topical fluoride refusal	51.5	0–1
Vaccine refusal	27.7	0–1
		
Concerns for side effects and safety		
Fluoride concern, mean (se)	1.22 (0.05)	0–3
Vaccine concern, mean (se)	1.62 (0.05)	0–3

Perceived severity of disease		
How bad if child got cavity preventable by fluoride, mean (se)	1.67 (0.05)	0–3
How bad if child got disease preventable by vaccine, mean (se)	0.68 (0.05)	0–3

Internet use for…		
Topical fluoride information	16.9	0–1
Vaccine information	20.8	0–1

Dental treatment/care		
Not ok with x-rays	25.7	0–1
Not ok with amalgam fillings	65.3	0–1
Not ok with composite fillings	17.9	0–1
No fluoride toothpaste use	9.5	0–1

a Note: all estimates based on 25 multiple imputed datasets of n = 361.

Though 51.5% of parents refused fluoride, only 27.7% reported refusing vaccinations. Fluoride concerns about side effects/safety were significantly lower (*p* < .05) than those for vaccines. Parents reported significantly lower perceived severity if their child developed a fluoride-preventable cavity versus a vaccine-preventable disease.

Fewer parents reported Internet use for information on fluoride (16.9%) versus vaccines (21.8%). For dental treatments, only 26% reported not being okay with dental x-rays, with more parents averse to amalgam (65.3%) than composite (17.9%) fillings, and <10% used non-fluoride toothpaste.

### 3.1 Correlations between measures

Table 2 reports bivariate (Pearson r) correlations for all fluoride and vaccine study measures. The correlations for construct-specific measures are demarcated with bold type.

**Table 2:** Bivariate correlations between topical fluoride-, vaccine-, and dental care-related variables.

	1	2	3	4	5	6	7	8	9	10	11
Refusal											
1. Topical fluoride refusal	1.00										
2. Vaccine refusal	**0.23**	1.00									

Concerns for side effects and safety											
3. Topical fluoride concerns	0.03	0.21	1.00								
4. Vaccine concerns	0.01	0.31	**0.65**	1.00							

Perceived severity of disease											
5. How bad if child got cavity preventable by topical fluoride	0.08	0.25	0.09	0.09	1.00						
6. How bad if child got disease preventable by vaccine	0.06	0.24	0.21	0.15	**0.39**	1.00					

Internet use for…											
7. Topical fluoride information	0.11	0.18	0.19	0.08	0.12	0.11	1.00				
8. Vaccine information	0.09	0.33	0.12	0.18	0.18	0.08	**0.41**	1.00			

Dental treatment/care											
9. Not ok with x-rays	−0.02	0.16	0.24	0.18	0.16	0.09	0.11	0.16	1.00		
10. Not ok with amalgam fillings	0.03	0.10	0.11	0.19	0.05	0.01	0.00	0.10	**0.27**	1.00	
11. Not ok with composite fillings	0.04	−0.03	0.13	0.04	−0.05	0.11	0.02	0.00	**0.37**	**0.19**	1.00
12. No fluoride toothpaste use	0.12	0.20	0.24	0.14	0.13	0.11	0.12	0.08	**0.17**	**0.11**	**0.05**

Note: estimates based on 25 multiple imputed datasets of n = 361. Bolded values indicate correlations between construct-specific items.

Topical fluoride and vaccine refusal are modestly correlated (r = 0.23), but stronger, positive correlations exist between concern for side effects/safety (r = 0.65), perceived severity of topical fluoride-preventable cavities and vaccine-preventable diseases (r = 0.39), and Internet use regarding topical fluoride- and vaccine-related information (r = 0.41).

The four dental treatment/care items show modest to small correlations overall, with the strongest correlations observed for not being okay with dental x-rays and both types of fillings: r = 0.27 for amalgam and r = 0.37 for composite. Furthermore, these items generally have small correlations with the attitude and behavior variables. However, attitudes towards x-rays and non-fluoride toothpaste use have comparatively higher—albeit modest—correlations with several fluoride and vaccine-specific items.

For all binary variables, supplementary analyses (not shown) using tetrachoric correlations revealed stronger magnitudes than the Pearson r values reported here.

### 3.2 Correlates of topical fluoride and vaccine refusal

Table 3 presents results of analyses assessing the extent to which these measures are associated with topical fluoride and vaccine refusal. For topical fluoride refusal, the first column reports unadjusted associations for each independent variable. Only two variables were significant associated with refusal: Internet use for topical fluoride information (prevalence ratio [PR] = 1.31) and not using fluoride toothpaste (PR = 1.41). Column 2 shows no significant associations for only vaccine-related factors (controlling for demographic covariates). In column 3, when all factors are included in the model, only not using fluoride toothpaste is significantly associated and shows a 36% higher likelihood of refusing topical fluoride refusal. Among the demographic factors (estimates not shown), income is the only variable associated with topical fluoride refusal—across all three models. In the full model, respondents reporting income >$40,000–$60,000 (versus greater than $60,000) were 45% less likely to refuse topical fluoride.

**Table 3:** Prevalence ratios (95% confidence intervals) for topical fluoride and vaccine refusal regressed on fluoride-, vaccination-, dental procedure/care-related factors; and sociodemographics.

	Topical fluoride refusal			Vaccine refusal		
	1	2	3	4	5	6
	Unadjusted	Vaccine items only; adjusted	Full model	Unadjusted	Fluoride/dental items only; adjusted	Full model
Concern for dangers and risks						
Topical fluoride concern	1.04 (0.93–1.15)		1.05 (0.89–1.23)	**1.42**⁎⁎⁎ (1.20–1.68)	**1.38**⁎⁎ (1.12–1.69)	1.02 (0.80–1.30)
Vaccine concern	1.01 (0.91–1.14)	1.00 (0.89–1.13)	0.97 (0.83–1.14)	**1.81**⁎⁎⁎ (1.49–2.20)		**1.57**⁎⁎ (1.19–2.07)

Perceived severity of disease						
How bad if child got cavity preventable by topical fluoride	1.10 (0.98–1.23)		1.07 (0.94–1.21)	**1.66**⁎⁎⁎ (1.35–2.01)	**1.45**⁎⁎ (1.17–1.78)	**1.29**⁎ (1.04–1.60)
How bad if child got disease preventable by vaccine	1.07 (0.96–1.19)	1.07 (0.96–1.20)	1.03 (0.91–1.15)	**1.45**⁎⁎⁎ (1.24–1.69)		1.18+ (0.99–1.40)

Internet use for…						
Topical fluoride information	**1.31**⁎ (1.05–1.64)		1.17 (0.90–1.53)	**1.90**⁎⁎⁎ (1.24–1.69)	**1.48**⁎ (1.04–2.12)	1.15 (0.75–1.76)
Vaccine information	1.21+ (0.97–1.51)	1.16 (0.92–1.46)	1.06 (0.82–1.38)	**2.84**⁎⁎⁎ (2.09–3.87)		**1.83**⁎⁎ (1.24–2.70)

Dental treatment/care						
Not ok with x-rays	0.96 (0.76, 1.22)		0.82 (0.64–1.07)	**1.68**⁎⁎ (1.20–2.35)	1.12 (0.79–1.59)	1.10 (0.79–1.53)
Not ok with amalgam fillings	1.07 (0.86–1.33)		1.04 (0.83–1.31)	1.46+ (0.98–2.16)	1.17 (0.75–1.80)	0.94 (0.61–1.45)
Not ok with composite fillings	1.10 (0.86–1.41)		1.20 (0.93–1.55)	0.88 (0.55–1.41)	0.86 (0.55–1.34)	0.90 (0.60–1.35)
No fluoride toothpaste use	**1.41**⁎⁎ (1.09–1.81)		**1.36**⁎ (1.02–1.89)	**2.25**⁎⁎⁎ (1.56–3.23)	1.25 (0.81–1.94)	1.34 (0.87–2.07)

Note: estimates based on 25 multiple imputed datasets of n = 361. Adjusted and full models control for all demographic variables listed in Table 1.

+ *p* < .10.

⁎ *p* < .05.

⁎⁎ *p* < .01.

⁎⁎⁎ *p* < .001.

For vaccine refusal, the unadjusted estimates (Column 4) indicate that all but concern about amalgam and composite fillings are significantly associated with higher likelihood of vaccine refusal. When only the fluoride and dental care (and demographic) items are included in the model (Column 5), higher likelihood of refusal is found among topical fluoride side effect/safety concern (PR = 1.38), severity of preventable disease (PR = 1.45), and Internet use for topical fluoride information (PR = 1.48). However, when all topical fluoride, vaccine, and dental care variables are included in the same model (Column 6), severity of preventable cavities is the only topical fluoride and dental care variable that is statistically significant (PR = 1.29); alongside vaccine concern and Internet use for vaccinations. Among sociodemographic factors, the only significant predictor was white (versus non-white) racial identity. In the full model, white respondents reported 62% higher likelihood of vaccine refusal.

Based on these series of models for both outcomes, the previously reported correlations, and sensitivity analyses (not shown), the full model results reported here do not appear to be due to collinearity or over-adjustment.

## 4 Discussion

Motivated by prior research reporting that topical fluoride and vaccine refusal are correlated, we investigated the degree to which topical fluoride, vaccine, and dental care attitudes and behaviors converge as well as the extent to which these factors cross-predict topical fluoride and vaccine refusal. Our findings indicate that (a) correlations between topical fluoride- and vaccine-specific concern, disease severity, and Internet-based information-seeking show evidence of converging constructs; however, (b) topical fluoride-related items are only predictive of vaccine refusal (not topical fluoride refusal) and these associations are mostly explained by their correlation with vaccine-related factors.

The clinical relevance of the study is that caregiver refusal of preventive care has become a problem that leads to greater disease burden for children and peers, potentially higher costs to the health care system, and preventable suffering (Chi and Basson, 2018). Most physicians and dentists lack training on how to effectively manage refusal behaviors, and tend to rely on fact-based paternalistic approaches, which are not effective for all caregivers who refuse preventive care (Hough-Telford et al., 2016). Previous research on vaccine hesitancy reported that presumptive approaches by clinicians during well child visits (e.g., the clinician saying to the parent “We have to do some shots, today”) lead to less parental refusal behaviors versus participatory approaches (e.g., the clinician *asking* the parent, “What would you like to do about shots?”) (Opel et al., 2013). However, the longer-term implications of such approaches are unclear, particularly in terms of balancing parent autonomy and child health outcomes (Opel et al., 2013; Brown et al., 2016).

Given this clinical significance, identifying factors that drive refusal of both of these preventive treatments is essential for creating more effective screening and intervention techniques for clinicians as well as complementary health promotion campaigns for public health agencies to pursue (Chi, 2017). Though we observed that vaccine and fluoride attitudes indicate some degree of overlap between refusal attitudes that may be predictive of vaccine refusal, topical fluoride refusal appears to be a unique behavioral phenomenon. These nuanced findings are not inconsistent with prior work that identified a correlation between topical fluoride and vaccine refusal (Chi, 2014)—a finding that is based on the same study data that we used here. Rather, our study advances that prior work by demonstrating that, like refusal behaviors for both of those preventive treatments, attitudes about them also overlap. However, the discordance that we observed for these correlated fluoride- and vaccine-focused measures in predicting *both* refusal outcomes, indicates that this degree of empirical overlap in attitudes is insufficient to explain the correlated refusal behaviors. Likewise, the dental treatment/care variables showed relatively weak correlations with fluoride attitudes, behavior, and refusal; thus suggesting that fluoride refusal is a distinct phenomenon among dental care treatments, except for non-fluoride toothpaste. Thus, refusing at-home and professional topical fluoride may be a way that caregivers are exerting control over health care decisions. The specific reasons for such refusal are unclear, but this underscores the value of dentists talking to these caregivers about the need for fluoride, especially in children at high-risk for dental disease.

Overall, it is important to recognize that, though the two refusal behaviors correlate, the magnitude of the correlation is relatively weak and thus highlights the fact that not all fluoride refusers are vaccine refusers and vice versa.

### 4.1 Limitations

Our study constitutes one of the first investigations to examine correlations between vaccine and topical fluoride attitudes and refusal, assessing several attitudinal and behavioral domains. Several limitations, however, need to be considered.

First, our data are from children sampled from three Washington state dental clinics. However, we collected data from a diverse set of clinics that treat children with public insurance, uninsured children, and those with private insurance. Furthermore, all of the study areas have fluoridated water (though not all of Washington state is fluoridated). Nevertheless, there is a possibility that individual families could be on well water, which may or may not have fluoride. To improve generalizability, future research should expand the types of clinics and communities from which caregivers who refuse preventive care are recruited.

Second, while topical fluoride refusal was based on chart records, vaccine refusal was self-reported. Because children within dental clinics only have dental records, any medical variables needed to be collected by survey instrument.

Third, our analysis did not account for a number of parental background factors that may contribute to such fluoride and vaccination attitudes and refusal. Future studies should examine parents' dental and medical history, notably fluoride treatment and vaccination receipt. In addition, our model did not include measures from all domains in the Health Belief Model and the Extended Parallel Process Model, a limitation that could be addressed through future mixed methods approaches.

## 5 Conclusion

In identifying how fluoride- and vaccine-related attitudes and behaviors overlap, but are associated with vaccine—but not fluoride—refusal, our study highlights the need for specialized research on the complex behavioral and social factors associated with refusal of preventive dental care. First, there is a need for detailed qualitative research to better understand why caregivers refuse various types of preventive care. Once the reasons are known, interventions tailored to specific reasons for refusal can be developed and refined. The eventual goal is to develop chairside screening tools that clinicians can use to identify caregivers who are likely to refuse and diagnostic tools that specify the reasons for refusal. These tools will then enable trained clinicians to intervene chairside with tailored approaches.

Furthermore, to the extent that fluoride and vaccine attitudes and Internet information-seeking are correlated (and non-fluoride toothpaste associated with fluoride refusal), it is necessary to further delineate the extent to which attitudes, behaviors, and refusal for topical fluoride, vaccinations, and other preventive treatments represent (a) isolated choices and behaviors, (b) a general risk calculus regarding benefits versus harms, or (c) a more latent health lifestyle composed of various parenting attitudes (Reich, 2016). Community and network-focused studies may help inform these questions given identified community clustering of vaccine refusal (Lieu et al., 2015).

Like vaccine refusal, much work remains in identifying factors that motivate fluoride refusal—to reduce the risk of caries and associated oral and other diseases and promote improved parental and general public understanding of the benefits of preventive treatments.

## Conflict of interest statement

No conflicts of interest exist for either author. This study was supported by the National Institute of Dental and Craniofacial Research of the National Institutes of Health (grants K08DE020856, L60MD003921, R03DE021439, and U54DE019346), the University of Washington Institute for Translational Health Sciences (grant UL1RR025014), the William T. Grant Foundation Scholars Program, and the Center for Advanced Study in the Behavioral Sciences (CASBS) at Stanford University. RMC received fellowship funding from the Killam Trusts while conducting this research. None of these funders had any role in the study design; collection, analysis, and interpretation of data; writing the report; and the decision to submit the report for publication.

## Financial disclosure

No financial disclosures were reported by the authors of this paper.
